# Characteristics of adverse reactions due to subcutaneous allergen immunotherapy applied between 2011-2021: Single center experience

**DOI:** 10.5578/tt.20239604

**Published:** 2023-12-04

**Authors:** Gürgün Tuğçe VURAL SOLAK, Kurtuluş AKSU, Yavuzalp SOLAK, Şenay DEMİR, Dilek ÇUHADAR ERÇELEBİ, Gözde KÖYCÜ BUHARİ, Sakine NAZİK BAHÇECİOČLU, İlkay KOCA KALKAN, Hale ATEŞ, Selma YEŞİLKAYA

**Affiliations:** 1 Division of Immunology and Allergic Diseases, Ankara Atatürk Sanatoryum Training and Research Hospital, University of Health Sciences, Ankara, Türkiye; 2 Division of Public Health, Şereflikoçhisar District Healt Directorate, Ankara, Türkiye

## Abstract

**ABSTRACT**

**
Characteristics of adverse reactions due to subcutaneous allergen immunotherapy applied between 2011-2021: Single center experience
**

**Introduction:** *The aim of this study was to elucidate the incidence of local, large local and systemic reactions after subcutaneus immunotherapy (SCIT) injections in our clinic and to determine the characteristic features of these adverse reactions.
*

**Materials and Methods:** *A total of 6000 SCIT injections administered to 163 patients between January 2011 and December 2021 were retrospectively evaluated. The study population consisted of patients with allergic rhinocon- junctivitis who underwent SCIT due to pollen, house dust mite or cat allergy, or patients who underwent SCIT due to venom allergy. Demographic charac- teristics of the patients, diagnoses, allergen sensitivities, immunotherapy pro- tocol applied, adverse reactions, and the characteristics of these reactions were recorded.
*

**Results:** *Totally, 163 patients with a mean age of 36.8 ± 12.7 years were enrolled in this research. Sex distribution was as follows: 55.2% (n= 90) were females. During the study, 218 allergic reactions were detected in 83 patients. The incidence of adverse reactions per injection was 3.6%. The probability of developing an adverse reaction in a patient during the entire subcutaneous immunotherapy was 53.9%. Of the adverse reactions that developed, 94 (43.1%, n= 47) were observed locally while 56 (25.7%, n= 40) were large local reactions, and 68 (31.2%, n= 30) were systemic. Incidence of adverse reactions per injection were 1.5%, 0.9%, and 1.1% for local reaction, large local reaction, and systemic reaction, respectively.
*

**Conclusion:** *The results of this analysis elaborated that subcutaneous immu- notherapy is a safe and tolerable treatment modality. However, before initiat- ing treatment, the benefits and risks should be evaluated. The risk of systemic reactions is quite low, but fatal anaphylaxis can occur, so physicians need to
*

*be aware of the potential risks.*

**Key words:** *Subcutaneus immunotherapy; allergen; safety; adverse reaction; side effect
*

**ÖZ**

**
2011-2021 yılları arasında uygulanan subkutan alerjen immünoterapisine bağlı gelişen yan etkilerin özellikleri: Tek merkez deneyimi
**

**Giriş:** *Bu çalışmanın amacı, kliniğimizde uygulanan subkutan immünoterapi (SKIT) enjeksiyonları sonrası gelişen lokal, geniş lokal ve sistemik reaksiyon insidansını ve bu advers reaksiyonların karakteristik özelliklerini belirlemektir.
*

**Materyal ve Metod:** *Ocak 2011 ve Aralık 2021 tarihleri arasında 163 hastaya uygulanan toplam 6000 SKIT enjeksiyonu retrospektif olarak değerlendirildi. Çalışma popülasyonu polen, ev tozu akarı ya da kedi alerjisi nedeniyle SKIT uygulanan alerjik rinokonjoktivitli hastalar ya da venom alerjisi nedeniyle SKIT uygulanan hastalardan oluşuyordu. Hastaların demografik özellikleri, tanıları, alerjen duyarlılıkları, uygulanan immünoterapi protokolü, advers reaksiyonlar ve bu reaksiyonların özellikleri kaydedildi.
*

**Bulgular:** *Bu araştırmaya yaş ortalaması 36,8 ± 12,7 olan toplam 163 hasta dahil edildi. Hastaların %55,2’si (n= 90) kadındı. Çalışma sırasında 83 hastada 218 alerjik reaksiyon tespit edildi. Enjeksiyon başına advers reaksiyon insidansı %3,6 idi. Tüm subkutan immü- noterapi uygulaması sırasında bir hastada advers reaksiyon gelişme olasılığı %53,9 idi. Gelişen advers reaksiyonların 94’ü (%43,1, n= 47) lokal, 56’sı (%25,7, n= 40) büyük lokal reaksiyonlar ve 68’i (%31,2, n= 30) sistemik olarak gözlendi. Enjeksiyon başına advers reaksiyon insidansı, lokal reaksiyon, büyük lokal reaksiyon ve sistemik reaksiyon için sırasıyla %1,5, %0,9 ve %1,1 idi.
*

**Sonuç:** *Bu çalışma subkutan immünoterapinin güvenli ve tolere edilebilir bir tedavi yöntemi olduğunu göstermektedir. Ancak tedavi- ye başlamadan önce yararları ve riskleri değerlendirilmelidir. Sistemik reaksiyon riski oldukça düşüktür, ancak ölümcül anafilaksi meydana gelebilir, bu nedenle hekimlerin potansiyel risklerin farkında olması gerekmektedir.
*

**Anahtar kelimeler:** *Subkutan immünoterapi; alerjen; güvenlik; advers reaksiyon; yan etki
*

## INTRODUCTION

For over a hundred years, since Noon published the first results of immunotherapy against hay fever in 1911, allergen immunotherapy (AIT) has been widely practiced by clinicians worldwide (1). AIT is the only treatment option that can be applied in treating allergic rhinoconjunctivitis (ARC), asthma, and venom hypersensitivity, which can change the course of the disease by targeting immune mechanisms (2-4). Allergen-specific antigens can be administered subcutaneously or sublingually. In subcutaneous immunotherapy (SCIT), the antigens determined to cause the patient’s clinical symptoms are administered to the patient for at least three years after the maintenance dose is reached, starting from very low doses and then increasing the dose at specified time intervals, in order to ensure that the patient can tolerate it in the next encounter with the antigen (2).

In several randomized controlled trials, it has been shown that SCIT improves ARC/asthma symptoms, reduces the need for pharmacotherapy in patients, and improves the quality of life in patients with ARC, asthma, and venom hypersensitivity (3,4). Despite its current benefits and acceptance as a reliable treatment method for allergic rhinoconjunctivitis, asthma and venom hypersensitivity, there are also adverse reactions related to SCIT (4,5). These adverse reactions can vary from local reactions (LR) to life-threatening anaphylaxis. LR manifests itself as redness, itching, and swelling at the injection site, and large lesions on the palm are considered large local reactions (LLR).

Systemic reactions (SR) range from pruritus, urticaria to anaphylaxis.

In the World Allergy Organization Grading System for Systemic Allergic Reactions (WAO 2017) classification, it is classified from mild (grade 1) to severe (grade 5) according to the severity of organ and system involvement (6). While LR can be treated with antihistaminic drugs, montelukast, cold applications, and injection in divided doses, SR is known to develop mostly within the first 30 minutes, and epinephrine applications may be required (2). For this reason, SCIT application should be applied by relevant specialists in experienced centers, after immunotherapy (IT) indication and patient selection, and the patient should be kept under observation for at least 30 minutes after the injection (2).

In the previous literature, the characteristic features and risk factors of adverse reactions after SCIT have been well documented. The presence of uncontrolled asthma, development of systemic allergic reaction in previous injections, application of immunotherapy in the season with high pollen load, delay in epinephrine administration, errors in allergen dosing and administration, insufficient observation time after injection, and out-of-hospital injection were stated as risk factors for adverse reactions (7-9).

The incidence of systemic reactions developing after SCIT has decreased over the years since allergists are aware of identifiable risk factors and take precautions accordingly (9). However, the distribution of adverse

reactions may differ by region, and data from a single country will not reflect the truth. Knowing the incidence of adverse reactions and risk factors will allow these treatments to be applied more reliably.

The aim of this retrospective analysis was to determine the incidence of LR, LLR and SR observed after SCIT injections with standardized commercial preparations and the characteristic features of these adverse reactions in patients diagnosed with ARC with/ without asthma or venom hypersensitivity.

### MATERIALS and METHODS

**Patient Population and Study Design**

This cross-sectional, retrospective study included patients who underwent allergen immunotherapy at Ankara Atatürk Sanatoryum Training and Research Hospital, Immunology and Allergic Diseases Clinic between January 2011 and December 2021. These patients were followed up with the diagnosis of ARC with/without asthma or venom hypersensitivity. The medical records of the patients were reviewed retrospectively. Data from 163 patients and 6000 injections were evaluated during the study.

Age, sex, diagnosis, and comorbidities were evaluated from the patient’s demographic information. In addition, the type of allergen that the patient was sensitive to (confirmed by the positivity of skin prick test and/or positivity of allergen-specific IgE), sensitization status for inhaled allergens (monosensitized/polysensitized), type of allergen used during immunotherapy (pollen, house dust mite, pollen + house dust mite, cat and venom), immunotherapy treatment protocol (conventional/ clustered), type of allergic reaction (LR, LLR, SR) and the development period (build-up phase/maintenance phase) and timing (immediate onset/delayed onset) of the allergic reaction were recorded.

The diagnosis and treatment of ARC, asthma, and venom hypersensitivity has been validated and regulated in accordance with the current Allergic Rhinitis and its Impact on Asthma (ARIA), Global Initiative for Asthma (GINA), and European Academy of Allergy and Clinical Immunology (EAACI) guidelines (2,10,11). Written informed consent was obtained from all patients before initiating SCIT.

The allergen sensitivity of the patients was determined by skin prick test positivity and/or specific serum IgE level measurement. The test was considered positive

if the induration diameter was over 3 x 3 mm. Specific serum IgE level ≥0.35 kUA/L was considered positive. All patients had a positive skin prick test against at least one aeroallergen and/or allergen sIgE≥ 0.35.

### Allergen Immunotherapy

SCIT was applied to patients whose symptoms persisted despite allergen avoidance and medical treatment, in line with the indications determined by the EACII guidelines on allergen ımmunotherapy (2). Allergen extracts such as allergovit (1000-10.000 TU/mL, Allergopharma, Hamburg, Germany) and alutard SQ (100-100.000 standardized quality units (SQ-U)/mL, ALK, Hørsholm, Denmark), etc., available in Türkiye, were used.

Immunotherapy injection times and doses were determined in accordance with the manufacturer’s recommendations and international guidelines (2,12,13).

Antihistaminic treatment was not routinely initiated in the patients at the beginning of immunotherapy. Immunotherapy dose was skipped in cases with ARC with/without asthma symptoms lost control, active infection, or recent bee sting.

The SCIT periods were classified as the build-up phase and the maintenance phase. Although injection doses varied according to the selected immunotherapy protocol, the maintenance dose was quickly achieved. In the maintenance phase, injections were administered every 4-6 weeks. SCIT was planned for at least three years for pollen, house dust mite and cat allergies, and five years for venom allergy. During the injections, similar protocols were applied with another center experienced in SCIT in our country (14).

Ankara Atatürk Sanatorium Training and Research Hospital, Immunology and Allergic Diseases Clinic nurses administered all injections. Subcutaneous injections were administered from the outer side of the arm with an angle of 45 to 90 degrees and a 26-ga (13 mm) insulin injector. After each injection, patients were kept under observation by allergists and nurses for 30 minutes. In addition, this situation was recorded in case of adverse reactions, and necessary treatment was administered. Each patient was examined before the injection, and their disease stability, current symptoms, and, if any, symptoms developed after the previous dose of IT were

questioned. Patients who developed delayed reactions were recorded, and the dose was administered accordingly. Resuscitation materials were kept ready for all emergencies.

Pollen and/or house dust, venom, and cat extracts were applied to the patients according to the allergen they sensitized. However, allergens were not mixed in the same injection.

### Classification of Adverse Reactions

Adverse reactions were classified according to their type and time of occurrence. Redness, itching, and swelling exceeding 5 cm and 10 cm in diameter at the injection site were classified as LR and LLR, respectively. If one or more system involvement developed, it was considered a systemic reaction. According to the WAO2017 modified Grading System for Systemic Allergic Reactions, the reactions were classified as grade 1, the lightest, and grade 5, the most severe (6). Reactions that developed in the first 30 minutes were defined as early onset, and those that developed after 30 minutes were defined as delayed onset.

All procedures followed were in accordance with the ethics standards of the responsible committee on human experimentation (institutional and national) and with the Helsinki Declaration of 1975, as revised in 2008. The study was carried out with the permission of Keçiören Training and Research Hospital Clinical Researches Ethics Committee (Date: 11.01.2022, Decision No: 2012-KAEK-15/2453).

### Statistical Analysis

Patient data collected within the scope of the study were analyzed with the IBM Statistical Package for the Social Sciences (SPSS) for Windows 20.0 (IBM Corp., Armonk, NY) package program. Frequency and percentage for categorical data and mean and standard deviation for continuous data was given as descriptive values. For comparisons between the groups, independent sample t-test was used for two groups, and Pearson's chi-square test was used to compare categorical variables. The results were considered statistically significant when the p-value was less than 0.05.

## RESULTS

A total of 163 patients, 90 (55.2%) females, were included in our study. The ages of the patients ranged

from 18 to 74, with a mean age of 36.8 ± 12.7. While the rate of patients followed up with the main diagnosis of ARC was 45.4% (n= 74), ARC + asthma in 31 patients (19.0%), venom allergy in 54 patients (33.1%), and cat allergy in four patients (2.5%). While monosensitization was observed in 65.0% (n= 106) of the patients, polysensitization was detected in 57 (35.0%) patients (Table 1).

While 53 patients (32.5%) had comorbidity, the most common comorbidity was asthma with 32.0% (n= 17), followed by allergic rhinitis in 28.3% (n= 15). Atopic dermatitis was found in two patients, urticaria in 10 patients, nasal polyps in nine patients, drug allergy in 10 patients, food allergy in four patients, oral pollen allergy syndrome in one patient, and contact dermatitis in one patient. None of the patients had a diagnosed mast cell disease.

The most common immunotherapy allergen type applied to the patients was pollen at 52.8% (n= 86), venom at 33.1% (n= 54), house dust mite at 8.6% (n= 14), followed by cat allergy at 2.5% (n= 4). House dust and pollen allergens were used together in five patients (3.1%). When immunotherapy treatment protocol of the patients was evaluated, conventional protocol was followed in 130 patients (79.8%), clustered protocol in 26 patients (15.9%), and conversion protocol from clustered immunotherapy to conventional immunotherapy was followed due to adverse reactions that developed in seven patients (4.3%) (Table 1).

While the total number of doses administered to the patients in the build-up phase was 2248, the total number of doses administered during the maintenance phase was 3752, and the total number of doses administered was 6000.

### Adverse Reactions

In total, 218 allergic reactions were detected in 83 patients. The incidence of allergic reactions per injection was 3.6%. The patient’s probability of developing an allergic reaction during the entire SCIT was 53.9%.

Of the adverse reactions that developed, 94 (43.1%, n= 47) were observed locally, while 56 (25.7%, n=

40) were large local, and 68 (31.2%, n= 30) were systemic. The incidence of adverse reactions per injection was 1.5%, 0.9%, and 1.1% for LR, LLR, and SR, respectively (Table 2).

**Table:** 

**Table 1.** Demographic and clinical characteristics of the patients with and without adverse reactions				
	**Total**	**Adverse reaction**		**Test**
	**n**	**No, n/%**	**Yes, n/%**	**p**
**Sex**				
Female	90	**55/61.1**	35/38.9	**0.004**
Male	73	28/38.4	45/61.6	
**Age**	163	33.0 (18-7)	36.5 (18-74)	0.170
		35.1 ± 11.1	38.6 ± 14.1	
**Atopy status**				
Monosensitized	106	**60/56.6**	46/43.4	**0.048**
Polysensitized	57	23/40.4	34/59.6	
**Main diagnosis**				
ARC	74	45/60.8	29/39.2	0.107
ARC + Asthma	31	15/48.4	16/51.6	
Venom hypersensitivity	54	21/38.9	33/61.1	
Cat hypersensitivity	4	2/50.0	2/50	
**Comorbidity**				
Yes	52	24/46.2	28/53.8	0.405
No	111	59/53.2	52/46.8	
**Allergen IT extract**				
Pollens	86	**56/65.1**	30/34.9	**0.001**
House dust mite	14	4/28.6	10/71.4	
Venom	54	21/38.9	33/61.1	
Cat	4	2/50.0	2/50.0	
House dust mite + Pollen	5	0/0.0	5/100.0	
**IT protocol**				
Conventional	130	69/53.1	61/46.9	**0.02**
Clustered	26	8/30.8	18/69.2	
Clustered to conventional	7	**6/85.7**	1/14.3

When we grouped adverse reactions according to the injection period, 135 (61.9%) were observed in the build-up phase, and the remaining 83 (38.1%) were observed in the maintenance phase. When we evaluated the adverse reactions according to the development time, 37 (16.97%) were immediate onset, and the remaining 181 (83.02%) were delayed onset (Table 3).

Adverse reactions developed in 83 (50.9%) patients.

29 (34.9%) subjects had 1 adverse reaction, 25

(30.1%) had 2, 8 (4.9%) had 3, 12 (14.5%) had 4, 2

(2.4%) had 5-6-8, 1 (1.2%) had 7-10-12 adverse reactions.

When we compared the development of adverse reactions with sex, the rate of adverse reaction

development in female patients was 61.1%, significantly higher than in male patients (p= 0.004). The rate of adverse reaction development in monosensitized patients was 56.6%, significantly higher than in polysensitized patients (p= 0.048). Adverse reaction rate (65.1%) was significantly higher in patients with pollen allergy (p= 0.001). The rate of adverse reaction development (85.7%) was significantly higher in patients switched from the clustered protocol to the conventional protocol (p= 0.02) than in patients treated with the conventional and clustered protocol. There was no significant relationship between the patient’s age, main diagnosis, and presence of comorbidity and adverse reaction development (Table 1).

**Table:** 

**Table 2.** Distribution and incidence of adverse reactions			
	**Local reaction (94)** **(n= 47)** **43.1%**	**Large local reaction (56) (n= 40)** **25.7%**	**Systemic reaction (68)** **(n= 30)** **31.2%**
Incidence of adverse reactions (per injection)	1.6%	0.9%	1.1%
	(94/6000)	(56/6000)	(68/6000)
Incidence of adverse reactions (per patient)	57.6%	34.3%	41.7%
	(94/163)	(56/163)	(68/163)

**Table:** 

**Table 3.** Distribution of adverse reactions during SCIT application							
		**Build-up phase**			**Maintenance phase**		
	**Immediate**	**Delayed**	**Total**	**Immediate**	**Delayed**	**Total**	**Total**
Local reactions	0	50	50	2	42	44	94
Large local reactions	1	36	37	1	18	19	56
Systemic reactions	20	28	48	13	7	20	68
Total reactions	21	114	135	16	67	83	218

### Systemic Adverse Reactions

Of the 68 systemic reactions, 70.6% (n= 48) developed during the build-up phase, and 29.4% (n= 20) developed during the maintenance phase. Of these reactions, 48.5% (n= 33) were immediate onset, and 51.5% (n= 35) were delayed onset. In addition, 42 (61.7%, n= 24) of the SR were observed as grade 1, while 13 reactions (19.1%, n= 9) were

grade 2, and 10 reactions (14.7%, n= 5) were grade

3, 2 reactions (2.9%, n= 1) were grade 4, and 1

reaction (1.4%, n= 1) was grade 5.

Twenty-two of the systemic reactions developed with pollen extract, four with house dust mite extract, 33 with venom extract and five with cat extract. The distribution of these reactions according to build-up and maintenance phases is shown in Figure 1.

SR development status was significantly higher in patients (57.1%) transferred from the clustered protocol to the conventional protocol (p= 0.025). There was no significant relationship between patients’ sex, age, atopy status, main diagnosis, comorbidity status, allergen types, and SR development status (Table 4).

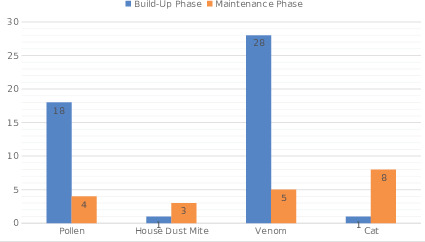

**Figure 1.** Numbers of systemic reactions according to allergen extracts.

**Table:** 

**Table 4.** Characteristic features of systemic reactions				
	**n**	**Systemic reaction** **Yes, n/% No, n/%**		**Test p**
**Sex**				
Female	90	16/17.8	74/82.2	0.819
Male	73	14/19.2	59/80.8	
**Age**	163	32.0 (18-71)	35.0 (18-74)	0.248
		34.9 ± 13.5	37.2 ± 12.6	
**Atopy status**				
Monosensitized	106	23/21.7	83/78.3	0.139
Polysensitized	57	7/12.3	50/87.7	
**Main diagnosis**				
ARC	74	14/18.9	60/81.1	0.527
ARC + Asthma	31	3/9.7	28/90.3	
Venom hypersensitivity	54	12/22.2	42/77.8	
Cat hypersensitivity	4	1/25.0	3/75.0	
**Comorbidity**				
Yes	52	10/19.2	42/80.8	0.852
No	111	20/18.0	91/82.0	
**Allergen IT extract**				
Pollens	86	14/16.3	72/83.7	0.715
House dust mite	14	3/21.4	11/78.6	
Venom	54	12/22.2	42/77.8	
Cat	4	1/25.0	3/75.0	
House dust mite + Pollen	5	0/0.0	5/100.0	
**IT Protocol**				
Conventional	130	22/16.9	108/83.1	**0.02**5
Clustered	26	4/15.4	22/84.6	
Clustered to conventional	7	**4/57.1**	3/42.9

Epinephrine was administered in three patients with adverse reactions, and epinephrine was administered for ten adverse reactions in these three patients (Table 5). SCIT was applied to all three patients who received epinephrine due to venom hypersensitivity.

Due to SR that developed in two patients, the transition from the clustered protocol to the conventional protocol was selected. Eight adverse reactions that developed were at baseline and immediate onset while two adverse reactions developed only delayed onset at baseline. No fatality was found in a total of 6000 immunotherapy doses administered.

## DISCUSSION

Since the beginning of the 20th century, it has been proven in placebo-controlled studies that allergen

immunotherapy has the potential to reduce allergic nasal symptoms and the use of rescue medication in the treatment of seasonal allergic rhinitis and also to have the potential to change the course of the disease. In addition, it is highly effective in treating perennial allergic rhinitis and IgE-dependent asthma and preventing anaphylaxis in case of venom hypersensitivity (15). However, AIT can cause serious systemic reactions ranging from local reactions to death (4,9,16-19).

In our study evaluating 6000 SCIT injections administered in our clinic for ten years, the incidence of adverse reactions was 3.6%, and systemic reactions were 1.1%. Adverse reactions that developed were mostly at build-up phase, delayed onset, and with pollen extracts. No fatality was found in 6000 SCIT injections administered.

**Table:** 

**Table 5.** Characteristic features of systemic reactions						
	**Reaction** **number**	**Age**	**Comorbidity**	**Administered** **allergen**	**Immunotherapy** **protocol**	**Property of reaction**
Patient 1	1	52	-	Venom	Clustered to	Build-up phase-Immediate onset-Grade 4
	2				conventional	Build-up phase-Immediate onset-Grade 3
	3					Build-up phase-Immediate onset-Grade 4
	4					Build-up phase-Immediate onset-Grade 3
Patient 2	5	21	Asthma	Venom	Conventional	Build-up phase-Immediate onset-Grade 5
	6					Build-up phase-Immediate onset-Grade 3
	7					Build-up phase-Immediate onset-Grade 2
	8					Build-up phase-Immediate onset-Grade 3
Patient 3	9	46	-	Venom	Clustered to	Build-up phase-Immediate onset-Grade 3
	10				conventional	Build-up phase-Delayed onset-Grade 2

According to the literature data, local reactions occur in 26-82% of patients who undergo SCIT and between 0.7% and 4% of injections (20-23). A systemic reaction develops in 0.1% to 0.8% of the patients (23,24). In a recent study in which 7372 injections have been evaluated in 323 patients, LR has been reported as 1.1% and SR 0.19% (23). In another study conducted in Türkiye, the incidence of adverse reactions and SR per injection has been reported as 2.6% and 1.3%, respectively (25). In 6000 SCIT injections evaluated in our study, 218 adverse reactions developed, with an incidence rate of 3.6%. LR developed 1.6% (n= 47), LLR 0.9% (n= 40), and SR 1.1% (n= 68). The LR and SR rates that developed are consistent with the literature data.

In our study, 55 (66.2%) of 83 patients who developed adverse reactions were females, and this rate was statistically significant (p= 0.004). In a study conducted in Portugal, adverse reactions during SCIT have been reported to. Be significantly higher in women (70%) (p= 0.002) (23). Moreover, publications state that adverse and systemic reactions develop more frequently in women (18,21,25-27). However, in our study, there was no significant sex difference between patients who developed systemic reactions. Many studies have found no significant difference between male and female patients with adverse reactions (19,25,28). Additional studies are needed since the hormone profile may be a risk factor in developing adverse reactions.

Previous studies have shown that adverse reactions due to immunotherapy develop more intensely in the build-up phase than in the maintenance phase (18,19,23,25,29). Baççıoğlu et al., in their study

evaluating the safety and compliance of SCIT, have reported that 75.2% of adverse reactions develop during the build-up phase and 24.8% during the maintenance phase (18). We also determined that adverse reactions occurred more frequently in the build-up phase (61.9%) than in the maintenance phase (38.1%). We found a similar distribution in all local, large local, and systemic reactions.

Dursun et al. have found that immediate onset (42.6%) reactions developed less frequently than delayed onset (57.4%) (25). In a multicenter study by Calderon et al. in Germany, France, and Spain, 41.3% of systemic reactions have occurred in the first 30 minutes, 8.3% between 30-60 minutes, 14.7% between 60-120 minutes, and 35.8%. improved after

120 minutes (19). In our study, 37 (17%) of 218 systemic reactions developed immediate onset, and 181 (83%) developed delayed onset similarly.

As a result of the questionnaire applied to North American allergists, it has been stated that 15% of systemic reactions develop after 30 minutes, 0.5% after 60 minutes, and only 5% of these reactions are considered severe reactions (30). In our study, epinephrine was administered in 10 systemic reactions in three patients. All of these reactions were in the build-up phase while eight reactions developed immediate onset, two were found to develop delayed onset.

Many studies have reported that systemic and fatal reactions mostly develop in the build-up phase and immediate onset, but rarely, delayed onset systemic reactions also develop (8,15,23,31). This suggests that patients should be observed for at least 30

minutes after the SCIT application.

We think that the observation period applied to the patients due to two delayed onset adverse reactions requiring epinephrine use can be extended by considering the underlying risk factors. In addition, prescribing an epinephrine auto-injector may be considered for patients with risk factors for a systemic reaction. Current guidelines do not recommend routinely prescribing epinephrine auto-injectors to patients undergoing SCIT, leaving this decision up to the treating physician (15).

Pollen extracts induce adverse reactions more frequently than mite extracts (19,25). In our study, the pollen group was significantly higher when we looked at the allergen extracts of the patients who developed adverse reactions like the literature data (p= 0.001). However, no statistically significant difference existed between the allergen extracts for patients who developed systemic reactions. Dursun et al. have reported that patients with pollen allergy experience more systemic reactions during the maintenance and pollen season than those with venom allergy (25), similarly, in the study of Rodriguez et al. where more than 1500 patients aged 18 years younger have been evaluated, 29 systemic reactions have been observed in 1.53% of the patients. It has been reported that patients with pollen allergy develop a higher systemic reaction compared to patients sensitive to house dust mites (32).

In the study of Nacaroğlu et al., although there is no difference in the incidence of adverse reactions between patients with mono allergen sensitization and those with multiple allergen sensitization, the risk of adverse reaction development has been found significantly higher in patients with a poly allergen in the allergen extract (2). Our current study did not observe any adverse reactions in our five patients who underwent immunotherapy with multi-allergens. Adverse reactions were significantly higher in monosensitized patients compared to polysensitized patients (p= 0.048).

Bronchial hyperreactivity is a serious risk factor for developing uncontrolled asthma adverse reactions (16,25). According to the North American survey results, asthma was found in two-thirds of the patients with grade 3 and 4 reactions (30). In line with these results, great care should be taken not to prescribe SCIT to patients with severe and uncontrollable asthma. In all patients diagnosed with asthma and

undergoing SCIT, questioning asthma symptoms and evaluation of lung function should be considered before each injection (9,15). When the data from 2008-2013 in North America and the data from 1990-2001 were compared, it was observed that there was a significant decrease in the number of injection-related adverse reactions among allergists (7,33). This trend has been associated with guideline recommendations emphasizing pre-injection screening or injection discontinuation in patients with severe, uncontrolled asthma, considered the most important risk factor for fatal reactions (34). In this study, asthma was not an increased risk factor for adverse and systemic reactions. This situation has been associated with the application of SCIT to patients with mild asthma, whose asthma symptoms are under control in accordance with the guidelines in our clinic, and the control of asthma symptoms before each SCIT injection.

Considering the increased risk of fatal reactions during the pollen peak season, it has been recommended not to increase immunotherapy doses during the pollen peak season in order to keep the systemic reaction risk at the lowest level (7,8). In a survey conducted in North America, severe grade 3 and grade 4 systemic reactions have developed significantly less in those who did not increase the dose during the pollen peak period (31). Although the period of the year with adverse reactions that developed in our study was not evaluated, the build- up phase of the patients who received immunotherapy with pollen extracts were applied outside the pollen season (between october and february), so precautions were taken against possible adverse reactions.

Fatal anaphylaxis is an extremely rare adverse reaction of SCIT. However, 2008 and 2018 data shows that fatal reactions occur in every 7.2 million, and life-threatening systemic reactions develop in every 160.000 injections (24). Additionally, no fatality has been observed in a study including 1098 pediatric patients with allergic rhinitis caused by a house dust mite allergy (35). No fatality was found in 6000 SCIT injections administered in our clinic within ten years. AR children caused by a house dust mite allergy.

In our study, it is very important to evaluate multiple injections with different allergens (pollen, house dust mite, cat and venom), SCIT injections with multiallergens, evaluate the relationship between

comorbidities and side effects, and examine 10-year real-life data.

Our results may be limited due to the single clinical experience. The limitations of the study are the lack of information on the duration of immunotherapy, the timing of the reactions that developed in the maintenance phase and the organs that develop systemic reactions (nasal, eye, respiratory, etc.).

## CONCLUSION

These results show that SCIT is a safe and tolerable treatment modality. However, before initiating SCIT treatment, the benefits and risks should be well evaluated. The risk of systemic reactions is very low, but fatal anaphylaxis can occur, so physicians should be aware of the potential risks. These practices should only be performed by clinicians familiar with these risk factors and able to manage treatment-related local/systemic reactions.

**Ethical Committee Approval:** This study was approved by Keçiören Training and Research Hospital Clinical Research Ethics Committee (Decision no: 2012- KAEK-15/2453, Date: 11.01.2022).

### CONFLICT of INTEREST

The authors declare that they have no conflict of interest.

## AUTHORSHIP CONTRIBUTIONS

Concept/Design: All of authors Analysis/Interpretation: All of authors Data Acqusition: All of authors Writing: All of authors

Clinical Revision: All of authors Final Approval: All of authors

## Notes

**REFERENCES**

1. *
  Noon L, Prophylactic inoculation against hay fever. The Lancet 1911: 177(4580): 1572-3. https://doi. org/10.1016/S0140-6736(00)78276-6*
2. *
  Roberts G, Pfaar O, Akdis CA, Ansotegui IJ, Durham SR, Gerth van Wijk R, et al. EAACI guidelines on allergen immunotherapy: Allergic rhinoconjunctivitis. Allergy 2018; 73(4): 765-98. https://doi.org/10.1111/all.13317*
3. *
  Roxbury CR, Lin SY. Efficacy and safety of subcutaneous and sublingual immunotherapy for allergic rhinoconjunc- tivitis and asthma. Otolaryngol Clin North Am 2017; 50(6): 1111-9. https://doi.org/10.1016/j. otc.2017.08.011*
4. *
  Dhami S, Zaman H, Varga EM, Sturm GJ, Muraro A, Akdis CA, et al. Allergen immunotherapy for insect venom aller- gy: A systematic review and meta-analysis [published correction appears in Allergy 2017; 72(10): 1590]. Allergy
  *

*
2017; 72(3): 342-65. https://doi.org/10.1111/all.13077*

5. *
  Sánchez-Borges M, Bernstein DI, Calabria C. Subcutaneous immunotherapy safety: Incidence per surveys and risk factors. Immunol Allergy Clin North Am 2020; 40(1): 25-39. https://doi.org/10.1016/j.iac.2019.09.001*
6. *
  Sánchez-Borges M, Ansotegui I, Cox L. World Allergy Organization grading system for systemic allergic reac- tions: It is time to speak the same language when it comes to allergic reactions. Curr Treat Options Allergy 2019: 6: 388-95. https://doi.org/10.1007/s40521-019-00229-8*
7. *
  Bernstein DI, Wanner M, Borish L, Liss GM; Immunotherapy Committee, American Academy of Allergy, Asthma and Immunology. Twelve-year survey of fatal reactions to aller- gen injections and skin testing: 1990-2001. J Allergy Clin Immunol 2004; 113(6): 1129-36. https://doi. org/10.1016/j.jaci.2004.02.006*
8. *
  Reid MJ, Lockey RF, Turkeltaub PC, Platts-Mills TA. Survey of fatalities from skin testing and immunotherapy 1985- 1989. J Allergy Clin Immunol 1993; 92(1 Pt 1): 6-15. https://doi.org/10.1016/0091-6749(93)90030-J*
9. *
  Bernstein DI, Epstein TG. Safety of subcutaneous allergen immunotherapy. Allergy Asthma Proc 2022; 43(4): 267-
  *

*
71. https://doi.org/10.2500/aap.2022.43.220035*

10. *
  Brożek JL, Bousquet J, Agache I, Agarwal A, Bachert C, Bosnic-Anticevichet S, et al. Allergic rhinitis and its ımpact on asthma (ARIA) guidelines-2016 revision. J Allergy Clin Immunol 2017; 140(4): 950-8. https://doi.org/10.1016/j. jaci.2017.03.050*
11. *
  Global Initiative for Asthma (GINA). Global Strategy for Asthma Management and Prevention. 2023. Available from: www.ginasthma.org (Accessed date: 17.07.2023).
  *
12. *
  Agache I, Lau S, Akdis CA, Smolinska S, Bonini M, Cavkaytar Ö, et al. EAACI guidelines on allergen immuno- therapy: House dust mite-driven allergic asthma. Allergy 2019; 74(5): 855-73. https://doi.org/10.1111/all.13749*
13. *
  Sturm GJ, Varga EM, Roberts G, Mosbech H, Bilò M, Akdis CA, et al. EAACI guidelines on allergen immunotherapy: Hymenoptera venom allergy. Allergy 2018; 73(4): 744-
  *

*
64. https://doi.org/10.1111/all.13262*

14. *
  Tepetam FM, CİHAN Ö, Özşeker ZF, Duman D, Saraç S. Can development of asthma and bronchial hyperreactivity be reduced by subcutaneous immunotherapy in adult patients with allergic rhinitis? Turk J Med Sci 2023; 53(3): 803-13. https://doi.org/10.55730/1300-0144.5643*
15. *
  Cox L, Nelson H, Lockey R, Calabria C, Chacko T, Finegold I, et al. Allergen immunotherapy: A practice parameter third update [published correction appears in J Allergy Clin Immunol 2011; 127(3): 840]. J Allergy Clin Immunol
  *

*
2011; 127(1 Suppl): S1-S55. https://doi.org/10.1016/j. jaci.2010.09.034*

16. *
  Amin HS, Liss GM, Bernstein DI. Evaluation of near-fatal reactions to allergen immunotherapy injections. J Allergy Clin Immunol 2006; 117(1): 169-75. https://doi. org/10.1016/j.jaci.2005.10.010*
17. *
  Aytekin G, Yıldız E, Çölkesen F, Arslan Ş, Çalışkaner A. Local and systemic reactions due to subcutaneous aller- gen immunotherapy: Our single-center 5-year experience. KSU Med J 2022; 17(1): 1-8. https://doi.org/10.17517/ ksutfd.839958*
18. *
  Baççıoğlu A, Kalpaklıoğlu AF, Poyraz M, Yalım SA, Dumanoğlu B, Alpağat G. Characteristics of adverse reac- tions and compliance in patients who underwent aller- gen-specific subcutaneous immunotherapy; ten-year real- life data. Asthma Allergy Immunology 2022; 20: 6-15. https://doi.org/10.21911/aai.632*
19. *
  Calderón MA, Vidal C, Rodríguez Del Río P, Just J, Pfaar O, Tabar AI, et al. European survey on adverse systemic reac- tions in allergen immunotherapy (EASSI): A real-life clini- cal assessment. Allergy 2017; 72(3): 462-72. https://doi. org/10.1111/all.13066*
20. *
  Tankersley MS, Butler KK, Butler WK, Goetz DW. Local reactions during allergen immunotherapy do not require dose adjustment. J Allergy Clin Immunol 2000; 106(5): 840-3. https://doi.org/10.1067/mai.2000.110468*
21. *
  Nelson BL, Dupont LA, Reid MJ. Prospective survey of local and systemic reactions to immunotherapy with pol- len extracts. Ann Allergy 1986; 56(4): 331-4.
  *
22. *
  Prigal SJ. A ten-year study of repository injections of aller- gens: Local reactions and their management. Ann Allergy 1972; 30(9): 529-35.
  *
23. *
  Coutinho C, Lourenço T, Fernandes M, Neto M, Lopes A, Spínola Santos A, et al. Subcutaneous immunotherapy with aeroallergens: Safety profile assessment. Eur Ann Allergy Clin Immunol 2022; 54(2): 77-83. https://doi. org/10.23822/EurAnnACI.1764-1489.194*
24. *
  Epstein TG, Murphy-Berendts K, Liss GM, Bernstein DI. Risk factors for fatal and nonfatal reactions to immuno- therapy (2008-2018): Postinjection monitoring and severe asthma. Ann Allergy Asthma Immunol 2021; 127(1): 64-69.e1. https://doi.org/10.1016/j.anai.2021.03.011*
25. *
  Dursun AB, Sin BA, Oner F, Misirligil Z. The safety of aller- gen immunotherapy (IT) in Turkey. J Investig Allergol Clin Immunol 2006; 16(2): 123-8.
  *
26. *
  Kartal O, Gulec M, Caliskaner Z, Musabak U, Sener O. Safety of subcutaneous immunotherapy with inhalant allergen extracts: A single-center 30-year experience from Turkey. Immunopharmacol Immunotoxicol 2015; 37(3):
  *

*
280-6. https://doi.org/10.3109/08923973.2015.1027918*

27. *
  Rank MA, Oslie CL, Krogman JL, Park MA, Li JT. Allergen immunotherapy safety: Characterizing systemic reactions and identifying risk factors. Allergy Asthma Proc 2008; 29(4): 400-5. https://doi.org/10.2500/aap.2008.29.3141*
28. *
  Schiappoli M, Ridolo E, Senna G, Alesina R, Antonicelli L, Asero R, et al. A prospective Italian survey on the safety of subcutaneous immunotherapy for respiratory allergy. Clin Exp Allergy 2009; 39(10): 1569-74. https://doi. org/10.1111/j.1365-2222.2009.03286.x*
29. *
  Nacaroglu HT, Erdem SB, Sumer O, Karaman S, Unsal Karkıner CS, Asilsoy S, et al. Local and systemic reactions to subcutaneous allergen immunotherapy: Ten years’ experience in a pediatric clinic. Ann Allergy Asthma Immunol 2016; 116(4): 349-53. https://doi.org/10.1016/j. anai.2016.01.015*
30. *
  Epstein TG, Liss GM, Berendts KM, Bernstein DI. AAAAI/ ACAAI subcutaneous immunotherapy surveillance study (2013-2017): Fatalities, infections, delayed reactions, and use of epinephrine autoinjectors. J Allergy Clin Immunol Pract 2019; 7(6): 1996-2003.e1. https://doi. org/10.1016/j.jaip.2019.01.058*
31. *
  Epstein TG, Liss GM, Murphy-Berendts K, Bernstein DI. AAAAI and ACAAI surveillance study of subcutaneous immunotherapy, year 3: What practices modify the risk of systemic reactions? Ann Allergy Asthma Immunol 2013; 110(4): 274-8.e1. https://doi.org/10.1016/j. anai.2013.01.015*
32. *
  Rodríguez Del Río P, Vidal C, Just J, Tabar AI, Sanchez- Machin I, Eberle P, et al. The European Survey on Adverse Systemic Reactions in Allergen Immunotherapy (EASSI): A paediatric assessment. Pediatr Allergy Immunol 2017; 28(1): 60-70. https://doi.org/10.1111/pai.12660*
33. *
  Epstein TG, Liss GM, Murphy-Berendts K, Bernstein DI. Risk factors for fatal and nonfatal reactions to subcutane- ous immunotherapy: National surveillance study on aller- gen immunotherapy (2008-2013). Ann Allergy Asthma Immunol 2016; 116(4): 354-59.e2. https://doi. org/10.1016/j.anai.2016.02.001*
34. *
  James C, Bernstein DI. Allergen immunotherapy: An updated review of safety. Curr Opin Allergy Clin Immunol 2017; 17(1): 55-9. https://doi.org/10.1097/ ACI.0000000000000335*
35. *
  Endaryanto A, Nugraha RA. Safety profile and issues of subcutaneous immunotherapy in the treatment of chil- dren with allergic rhinitis. Cells 2022; 11(9): 1584. https://doi.org/10.3390/cells11091584*

